# An Evaluation of the Acoustic Activity Emitted in Fiber-Reinforced Concrete Under Flexure at Low Temperature

**DOI:** 10.3390/s25092703

**Published:** 2025-04-24

**Authors:** Omar A. Kamel, Ahmed A. Abouhussien, Assem A. A. Hassan, Basem H. AbdelAleem

**Affiliations:** 1 Kinectrics Inc., 393 University Ave, Toronto, ON M5G 1E6, Canada; oalaaeldein@mun.ca; 2 Department of Civil Engineering, Faculty of Engineering and Applied Science, Memorial University of Newfoundland, St. John’s, NL A1B 3X5, Canada; ahassan@mun.ca; 3 Faculty of Engineering, Cairo University, Cairo University Rd, Oula 3725121, Egypt; bhaa18@mun.ca

**Keywords:** fiber-reinforced concrete, passive acoustic emission analysis, four-point flexural test, crack classification, cold temperatures

## Abstract

This study investigated the changes in the acoustic emission (AE) activity emitted in fiber-reinforced concrete (FRC) under flexure at two temperatures (25 °C and −20 °C). Seven concrete mixtures were developed with different water-binder ratios (w/b) (0.4 and 0.55), different fiber materials (steel fiber (SF) and synthetic polypropylene fiber (Syn-PF)), different fiber lengths (19 mm and 38 mm), and various Syn-PF contents (0%, 0.2%, and 1%). Prisms with dimensions of 100 × 100 × 400 mm from each mixture underwent a four-point monotonic flexure load while collecting the emitted acoustic waves via attached AE sensors. AE parameter-based analyses, including *b*-value, improved *b*-value (*Ib*-value), intensity, and rise time/average signal amplitude (RA) analyses, were performed using the raw AE data to highlight the change in the AE activity associated with different stages of damage (micro- and macro-cracking). The results showed that the number of hits, average frequency, cumulative signal strength (CSS), and energy were higher for the waves released at −20 °C compared to those obtained at 25 °C. The onset of the first visible micro- and macro-cracks was noticed to be associated with a significant spike in CSS, historic index (*H (t)*), severity (*S_r_*) curves, a noticeable dip in the *b*-value curve, and a compression in bellows/fluctuations of the *Ib*-value curve for both testing temperatures. In addition, time and load thresholds of micro- and macro-cracks increased when samples were cooled down and tested at −20 °C, especially in the mixtures with higher w/b, longer fibers, and lower fiber content. This improvement in mechanical performance and cracking threshold limits was associated with higher AE activity in terms of an overall increase in CSS, *S_r_*, and *H (t)* values and an overall reduction in *b*-values. In addition, varying the concrete mixture design parameters, including the w/b ratio as well as fiber type, content, and length, showed a significant impact on the flexural behavior and the AE activity of the tested mixtures at both temperatures (25 °C and −20 °C). Intensity and RA analysis parameters allowed the development of two charts to characterize the detected AE events, whether associated with micro- and macro-cracks considering the temperature effect.

## 1. Introduction

The properties of materials generally (specifically concrete) change drastically when the temperature drops from ambient to subnormal temperatures [1,2]. Previous studies reported an increase in flexure strength, elastic stiffness, and compressive strength at low temperatures [3,4]. Despite the aforementioned beneficial effects, concrete is shown to be more brittle [4,5]. Sloan [6] studied the seismic behavior of reinforced concrete members at low temperatures and reported that decreasing temperature to −40 °C led to a sudden failure once they reached the maximum compressive strength indicating a brittle failure. Various solutions were proposed to improve concrete’s brittle behavior, especially at low temperatures. One of the most effective approaches is designing concrete with randomly dispersed discontinuous fibers [7,8].

By including fibers, concrete’s intrinsic flaws, such as poor post-cracking ductility, low tensile strength, and low deformation capacity can be controlled [9,10]. Hence, cementitious materials will be damaged by numerous narrow cracks, thereby reducing the risk of catastrophic failure [11,12,13]. Ismail and Hassan [14] investigated the effect of including various lengths and contents of steel fibers on the mechanical properties and impact resistance of rubberized concrete mixtures. The results revealed that increasing steel fiber content up to 1% increased the compressive strength, splitting tensile strength, flexure strength, and impact resistance by 1.07, 1.93, 1.75, and 4 times, respectively, to that of a control mixture without fiber. The mechanical behavior of fiber-reinforced concrete (FRC) is dependent on a number of factors, including the water-binder (w/b) ratio and fiber type, volume fraction, length, strength, and stiffness [15,16,17].

Among several techniques used to assess the concrete’s performance, an acoustic emission (AE) analysis is a real-time non-invasive technique that has been used by many researchers in FRC applications [18,19,20,21,22,23]. AE refers to sound waves produced when a material undergoes cracking. The characteristics of the generated AE wave (e.g., counts, the number of hits, amplitudes, rise time, duration, frequency, signal strength, and energy) are proportional to the size of crack growth, which can provide valuable information regarding cracking levels [24,25,26]. An AE analysis can take the form of parameter-based or waveform-based AE approaches. A waveform-based AE analysis can offer more information than a parameter-based analysis regarding source identification and signal characterization [27], yet a parameter-based analysis, such as *b*-value, improved *b*-value (*Ib*-value), intensity, and rise time/average signal amplitude (RA) analysis approaches, may provide quantitative results that can be used to evaluate the extent of damage in concrete structures.

The *b*-value and intensity analyses performed by Abouhussien and Hassan [18] on the AE signal amplitudes emitted under flexure in fiber-reinforced self-consolidated concrete allowed for the early detection of two stages of damage (micro- and macro-cracking) prior to failure. In addition, analyzing the change in RA values and average frequency (AF) allowed Aggelis, Soulioti [20] to highlight the effect of chemically treated and untreated steel fibers on the fracture mechanism of FRC under four-point flexure tests. An RA analysis allowed Liu, Li [28] to monitor the mechanical performance of crumb rubber basalt fiber-reinforced concrete under a three-point flexure test.

Concrete mixtures with different microstructures and mechanical properties produce different types of acoustic activity when they undergo cracking [29]. Consequently, factors that affect concrete microstructure, such as cold temperatures, are anticipated to change the behavior of AE waves emitted in concrete mixtures and should be investigated. The sensitivity of the AE monitoring technique to the changes in the concrete microstructure was examined in this study. Whilst the behavior of AE waves emitted in FRC under flexure at ambient room temperature is well documented [18], the literature lacks information regarding the change in acoustic activity at subfreezing temperatures. The ultimate goal of this study is to fill this gap and to assess the effect of different w/b and fiber types, lengths, and contents on AE wave propagation at low temperatures reaching −20 °C. The selected low temperature value of −20 °C considered in this study was based on the average operating temperatures during the winter months in North America. Furthermore, the study aims to develop criteria to characterize the micro- or macro-cracking stages considering the temperature effect and the aforementioned mixture design variables.

## 2. Research Significance

AE waves accompanying the nucleation of concrete cracks can provide valuable information about the damage if properly collected and analyzed. Any change in transfer medium microstructure caused by factors such as the concrete type and the exposure to cold temperature can affect the behavior of AE waves. As a result, this study investigates the behavior of the AE waves emitted in FRC under flexure at low temperature. In addition, this paper studied the effect of varying the w/b, fiber materials (steel or synthetic polypropylene), fiber contents (0%, 0.2%, and 1%) and fiber lengths (19 mm and 38 mm), and testing temperatures (−20 °C and 25 °C) on the behavior of the AE waves. This study also developed classification charts based on intensity and RA analysis parameters that consider temperature effect to evaluate the cracking behavior of FRC. This study is anticipated to enrich the integration of the AE analysis method as a non-destructive technique for FRC in cold regions.

## 3. Experimental Program

### 3.1. Concrete Mixture Development

The trail mixture stage targeted a class of SCC mixtures having a slump flow diameter of 700 ± 50 mm, meeting the SCC conformity criteria given by the EFNARC (2005) [30]. Several trial mixtures were performed to determine the minimum w/b, total binder content, optimum type and content of supplementary cementing materials (fly ash (FA) or metakaolin (MK)), and the type and content of fibers required to develop mixtures M1, M6, and M7, meeting the acceptable fresh properties limits of SCC according to the EFNARC (2005) [30] without overdosing the high-range water-reducing admixtures (HRWRAs). Meanwhile, mixtures M2–M5 were adopted from a previous study performed by the authors [23]. The optimum results of the seven concrete mixtures are listed in Table 1. Since the characteristics of the acoustic waves are related to the mechanical properties and microstructure of the damage source [29], two non-fibrous SCC mixtures (M1 and M2) were developed with different w/b (0.55 and 0.4, respectively) to investigate the effect of turning a high amount of water into ice on the behavior of the acoustic waves when samples are cooled down and tested at −20 °C. The variation in the w/b ratios is expected to have an impact on the microstructure of concrete and the overall flexural behavior of concrete at low temperatures.

Mixture designation was as follows:

- M500-0.55 w/b means a concrete mixture with a binder content of 500 kg/m^3^ and 0.55 water–binder ratio.
- M550-SynF38 means a concrete mixture with a binder content of 550 kg/m^3^ and reinforced with synthetic fibers 38 mm in length.

**Figure 1:**
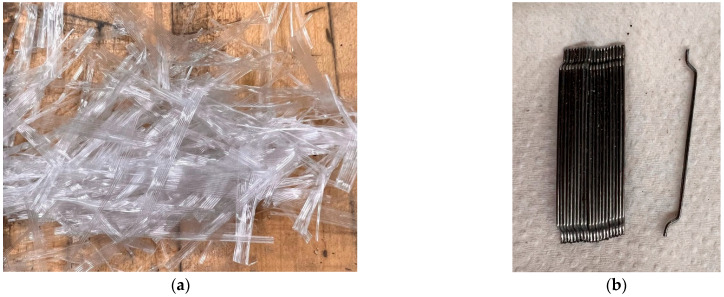
Pictures of the fibers used: (**a**) 38 mm polypropylene synthetic fibers, (**b**) 35 mm single hooked-end steel fibers.

In addition, five concrete mixtures (M3–M7) with 550 kg/m^3^ binder content were developed as follows: control non-fibrous SCC mixture (M3); two SCC mixtures with 0.2% Syn-PF but different lengths (19 mm and 38 mm) (M4 and M5, respectively); one SCC mixture reinforced with 0.2 SF (35 mm) (M6); and one SCC mixture with 1% Syn-PF 38 mm length (see Figure 1). These mixtures are aimed at evaluating the effect of changing the mixture design parameters of concrete on the flexural behavior and the corresponding AE response. The geometrical and mechanical characteristics of these fibers are described in Table 2. Fibers were selected based on their commercial availability. Two percentages of fibers (0.2% and 1%) were selected in this investigation to study the effect of the increased activity of fibers’ pull-out and breakage on the AE activity. In the SCC mixtures, 0.2% was found to be the maximum possible dosage of fibers that satisfied the requirements of fresh properties for SCC [30], while the 1% fibers in normal vibrated concrete (M7) was the maximum percentage of fibers that ensured a homogeneous mixture with no fiber balling [31].

### 3.2. Material Properties

General-use Canadian Portland cement (type GU) was used in all mixtures, which was in compliance with ASTM Type Ι [32], and had a specific gravity of 3.15. Fly ash (FA) and metakaolin (MK) were used as supplementary cementing materials to enhance the mixture suspension. FA and MK had specific gravity of 2.5 and 2.38, respectively. The physical and chemical properties of all cementitious materials are listed in Table 3 and Table 4. Both coarse aggregate (C.A.) and fine aggregate (F.A.) used in this investigation had a specific gravity of 2.6 and water absorption of 1%. The coarse-to-fine aggregate ratio was taken at 0.7 in all mixtures to achieve acceptable fresh properties of the SCC mixtures. The W/B ratio was kept constant at 0.4 in all mixtures except for mixture M2, where a W/B of 0.55 was used to study the effect of higher W/B (that will turn into ice after freezing) on the flexure performance and the characteristics of the AE waves at −20 °C. To obtain successful SCC mixtures (in terms of fresh properties), a high-range water-reducing admixture (HRWRA) was added to all SCC mixtures to achieve the desired slump flow of 700 ± 50 mm, as per ASTM C C1611 [33]. The HRWRA used was similar to that described in ASTM Type F [34] and had a specific gravity of 1.2 and a pH level of 9.5.

### 3.3. Specimen Details and Preparation

**Figure 2:**
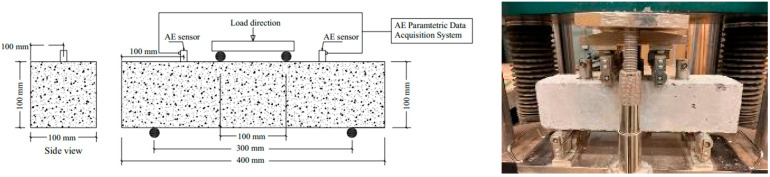
Four-point load flexure setup, AE sensors’ locations and samples’ positioning.

To perform compressive strength according to ASTM C39 [24] and flexure test according to ASTM C78 [25], six cylinders (100 mm diameter and 200 mm height) and four prisms (100 × 100 × 400 mm) were cast from each mixture (see Figure 2). Samples were kept at ambient room temperature for 24 h before demolding. Then, the samples were de-molded and kept in a moist curing room at 25 °C for 28 days to reach the target strength. The moist curing room was used to maintain relative humidity in the range of 80% to 85% throughout the curing period. After reaching maturity, samples were divided into two groups. Each group consisted of three cylinders and two prisms. For each mixture, one group was tested at room temperature (25 °C), and the other group was kept in a freezer for 48 h to reach a steady-state temperature of −20 °C before testing [26].

### 3.4. Four-Point Flexure Load Setup

A Tinius Olsen universal hydraulic machine was used to perform the four-point flexure test, as shown in Figure 2. The machine conducted the test on a deflection control mode at a constant displacement rate of 0.2 mm/min until failure according to ASTM C78 [25]. The load resisted by the specimens with time was recorded with the aid of a data-acquisition system.

### 3.5. AE Monitoring Setup

All prisms were tested under ongoing AE monitoring throughout the flexure test via the two attached AE sensors shown in Figure 2. Those piezoelectric sensors had an integral preamplifier model R6I-AST [27]. This type was suitable for the test due to its high sensitivity and low resonant frequency (55 kHz), which made it more convenient to monitor failures in many previous applications, such as metals, fiber-reinforced polymers, and concrete structures [6,27,28]. The two sensors were attached to the surface of each prism using a two-part epoxy adhesive. The exact locations of the sensors are shown in Figure 2. The attached sensors were connected to cables leading to a four-channel AE data acquisition system and AEwin signal processing software (AEwin for USBTM software, Version E3.32) provided by the manufacturer (Mistras Group) [29], which was used to record the signals emitted throughout the tests.

## 4. AE Data Filtering

### 4.1. Amplitude-Duration AE Data Filtering

To eliminate noise data and vast wave reflections within the boundaries of samples, the data acquisition system had built-in analog and digital filters, as shown in Table 5.

### 4.2. Post-Testing Amplitude-Duration AE Data Filters

Moreover, an amplitude-duration-based filter (Swansong II filter) was applied post-testing on the data collected during the four-point flexure test, as can be seen in Table 6. This filter is based on the concept that the real high-amplitude AE signals are typically associated with long values of signal duration and vice versa. The limits of this filter were identified by means of the visual inspection of the resulting AE signals, as can be seen in Table 5. This filter was effective in isolating the noise resulting from the contact between loading heads and specimens, and it was successfully adapted in many previous AE-based studies [6,31,35]. 

## 5. AE Analysis Methods

In the parameter-based approach, any signal is reduced to a few sets of parameters, and the evolution of these parameters with time or any external parameter (e.g., the applied load or crack width development) is investigated to establish a specific correlation that might exist between damage progression and the investigated AE parameters. This paper adopted a set of parameter-based analyses as follows:

### 5.1. b-Value Method

The *b*-value analysis method was first used in seismic waves generated by earthquakes and has been recognized extensively by seismologists for quantifying seismicity [35,36]. AE waves are similar to seismic waves, and the *b*-value method can also be applied to the AE field. The Gutenberg–Richter (GR) law [37] of seismicity can be modified for the AE analysis as follows:

(1) $$b=\left(a-\log N_{A}\right)/\log A_{dB}$$

where N_A_ is the number of AE events with amplitudes greater than A_dB_ precedent to a specific event, A_dB_ is the peak amplitude of the AE event in decibels, a is the intercept along the log N-axis, and b is the slope of the regression line, also called the *b*-value of these AE events. From Equation (1), a bigger *b*-value can be obtained when micro-cracks begin to form. In contrast, a smaller *b*-value can be obtained when macro-cracks develop.

### 5.2. Ib-Value Method

Shiotani et al. [38] proposed the improved *b*-value (*Ib*-value) method after it had been applied successfully to geotechnical and concrete materials [39,40]. The *Ib*-value has been defined by utilizing statistical values of AE events, and it can be calculated as follows:

(2) $$Ib=\frac{logN\left(µ-\alpha \sigma \right)-logN(µ+Ѱ\sigma )}{(\alpha +Ѱ)\sigma }$$

Ib-value is usually calculated for a group of events, where μ and σ are the mean values and standard deviation of the amplitude distribution of the AE event group, respectively, and α and Ѱ are user-defined constants, which would represent the coefficients of the lower and upper limits of the amplitude. It is understood that the *Ib*-value is a transient feature that is updated with each new hit recorded during the fracture process. It should be sensitive enough to even small fracturing events, so the population N is usually set to 100 recent hits [41].

### 5.3. AE Signal Intensity Analysis

The damage of concrete structures can be characterized using the intensity analysis of the AE signals. Intensity analysis involves the plot of historical index (HI) and log severity (log *S_r_*), both of which are based on the AE signal strength [42]. AE signal strength takes the amplitude and duration into account, so it is the most effective parameter to determine the trend of the AE data [43].

HI measures changes in the signal strength throughout the loading process. HI at a specific time (*H (t)*) is calculated using the following equation [44]:

(3) $$H\;\left(t\right)=\frac{N}{N-K}\frac{\;\sum_{i=K+1}^{N}S_{oi}}{\sum_{i=1}^{N}S_{oi}}$$

where N is the number of hits up to time t, $S_{oi}$ is the signal strength of the specific event, and K is an empirical parameter that depends on the number N and can be calculated as shown in Table 7. HI can be used to highlight the change curve in the cumulative signal strength versus time. This method can determine the onset of new damages for the specimen or structure.

The severity index (*S_r_*) is defined as the average signal strength for the J hits that have the largest numerical value of signal strength [45]. It can be calculated using Equation (4):

(4) $$S_{r}=\sum_{i=1}^{J}\frac{S_{oi}}{J}$$

where $S_{oi}$ is the signal strength of a specific event and *J* is an empirical constant related to the material for concrete structures, as shown in Table 7. Therefore, *S_r_* refers to the average strength of the 50 signals with the largest peak amplitudes of signal strength. 

### 5.4. Average Frequency (AF) Versus Rise Time/Amplitude (RA) Analysis

According to RILEM [46], the average frequency (AF) is defined as the number of counts in a hit divided by the duration of the signal, and RA-value is calculated from the rise time divided by the amplitude. The AF is measured in kHz, while the value of RA is measured in μs/V. The two parameters can be calculated based on the following equations:

AF = AE ringdown counts/duration time(5)

RA = the rise time/amplitude(6)

**Figure 3:**
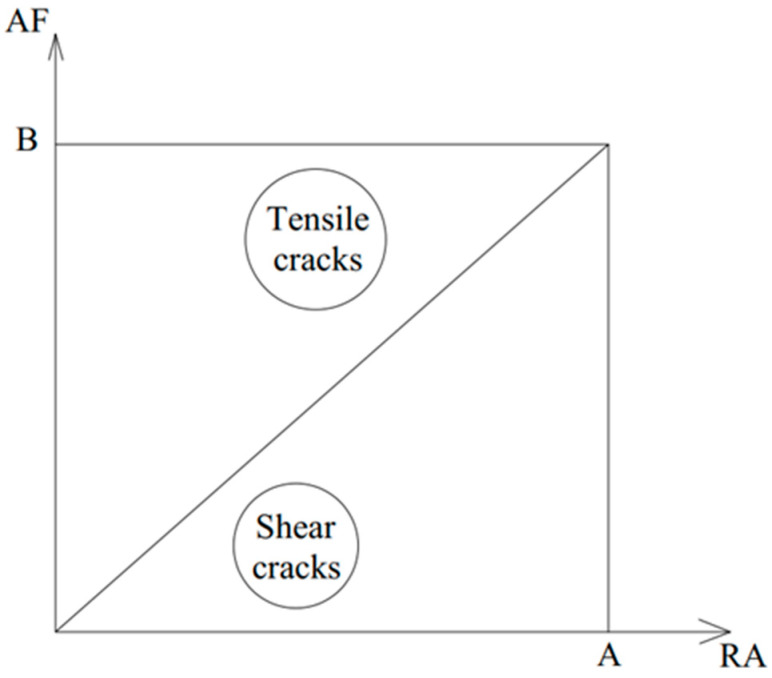
Typical crack classification based on the relationship between the AF and RA values [47].

Based on the relationship between the AF and RA value established by Ohtsu et al. [47], the cracks can be classified into tensile cracks and shear cracks (Figure 3). A shear crack has occurred when the AE signal has high RA and low AF values. Meanwhile, tensile cracks have occurred when the AE signal has low RA and high AF values [43]. See Table 8.

## 6. Results and Discussion

Table 9 lists the compressive and flexure strength for all mixtures at both temperatures (25 °C and −20 °C). The onset times and loads of both micro- and macro-cracking stages that were noticed visually and detected by the AE analysis are listed in Table 10 and Table 11. All the results are discussed in further details in the following sections.

### 6.1. Micro- and Macro-Crack Stages’ Onset Indicators in Terms of AE Analysis Parameters

**Figure 4:**
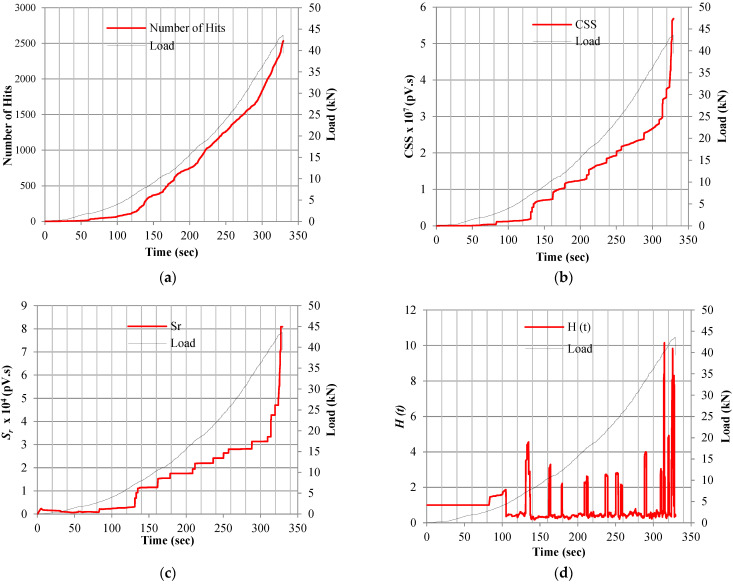

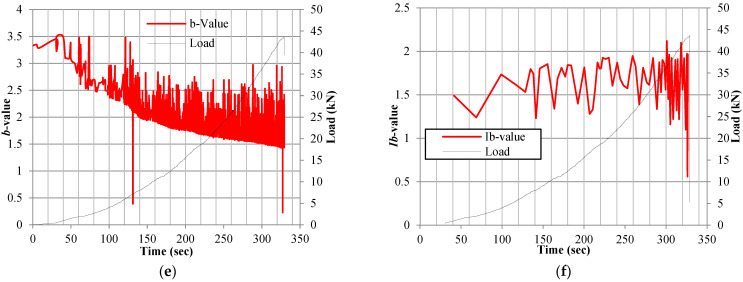
The change in AE parameters throughout flexure test versus applied load on sensor-2 sample-1 mixture M550-SynF19 tested at −20 °C: (**a**) number of hits, (**b**) CSS, (**c**) *S_r_*, (**d**) *H (t)*, (**e**) *b*-value, and (**f**) *Ib*-value.

The variation in the AE waves’ parameters collected by the monitoring setup, such as number of hits, CSS, signal amplitudes, and the results of the various parameter-based AE analyses, were related to the visually noticed micro- and macro-cracks. Figure 4 shows the variation of the AE activity parameters emitted during the flexure test by sensor-2 for sample-1 of mixture M550-SynF19 when tested at −20 °C along with flexure load-time curve applied during the test. The comparison between the applied load and the corresponding AE parameters shown in Figure 4 indicated that the studied AE parameters are sensitive to the cracking behavior of the tested specimen throughout the test till failure. An overall increase was noticed in the values of the number of hits, CSS, and *S_r_* indicating an increased AE activities due to the initiation of micro-cracks and propagation of macro-cracks. On the other hand, the values of the *H (t)*, *b*-value, and *Ib*-value fluctuated throughout the test time as the AE activities were increased since these parameters are more sensitive to the extent of damage compared with the number of hits, CSS, and *S_r_*. As shown in Figure 4a, the number of hits curve followed and overall increasing pattern with no noticeable changes along with the flexure test. Hence, the change in the number of hits cannot be used as a cracking onset indicator. Meanwhile, there was a noticeable spike in CSS, *S_r_*, and *H (t)* curves after 132 s, and this spike was recorded 3 s before visually noticing the first micro-crack. In addition, the *b*-value curve kept fluctuating with a decreasing pattern, and there was a noticeable dip at the same detected time (132 s). In addition, the *Ib*-value curve kept fluctuating with no noticeable pattern change prior to the micro-crack onset stage.

Previous researchers related the sharp variations in the CSS, *S_r_*, *H (t)*, *b*-value, and *Ib*-value curves to different damage mechanisms [29,38,41,52]. In particular, a previous study mentioned that the formation of the first micro-crack was accompanied by the first slope change in the *S_r_* curve versus time and the first drastic dip in *b*-value and *Ib*-value curves versus time [34]. It should be highlighted that the AE waves’ parameter analysis allowed for the detection of micro-crack onset earlier than the visual inspection, as shown in Table 10. On average, at −20 °C, the AE monitoring detected the micro-cracks 2 secs earlier for mixture M500-0.55w/b, 2 secs earlier for mixture M500-0.4w/b, 1 sec earlier for mixture M500-control, 7 secs earlier for mixture M550-SynF19, 5 secs earlier for mixture M550-SynF38, 9 secs earlier for mixture M550-1%SynF38, and 8 secs earlier for mixture M550-SF35. This earlier alarm could be due to the formation of invisible micro-cracks inside the tested samples; meanwhile, the visual inspection can only detect the micro-cracks developed on the surface.

Similarly, as shown in Figure 4b–d, a sharp spike in CSS, *H (t)*, and *S_r_* curves was noticed after approximately 324 s when the sample reached a load of 43.45 KN before failure. In addition, a significant dip in the *b*-value coincided with the formation of a visually noticeable macro-crack near the midspan of the tested sample after the previously mentioned time (324 s). In addition, the bellows of the *Ib*-value curve fluctuations became closer and higher prior to failure (macro-crack onset). It should be mentioned that almost all other tested samples failed after showing similar AE change patterns before failure. The higher values of the AE events recorded at this stage can be attributed to the further propagation of micro-cracks, which eventually led to the creation of micro-cracks. Therefore, monitoring previously mentioned AE waves’ parameters can be considered as indicators to the onset of micro- and macro-cracking stages.

### 6.2. Cold Temperature Effect on AE Activity Parameters

Overall, the mechanical performance of the tested concrete mixtures in terms of compressive and flexural strength was enhanced and the micro- and macro-crack load and time threshold increased along with decreasing the samples’ temperature to −20 °C (see Table 9, Table 10 and Table 11). The AE monitoring setup detected the onset of micro-cracks when the loads reached approximately 27–36% and 33–41% of the failure loads when samples were tested at 25 °C and −20 °C, respectively. The results of the first cracking loads, failure loads, and the corresponding studied AE parameters at both the times of micro- and macro-cracking detection for all tested samples are summarized in Table 10 and Table 11.

For instance, mixture M4 reached an average compressive strength of 78.63 MPa and an average flexure strength of 14.84 MPa when samples were tested at 25 °C. Both values increased by 15.4% and 24.9% when samples were cooled down and tested at −20 °C. The same mixture witnessed the formation of the first micro-crack at 7.41 KN when tested under flexure at −20 °C, and this value was 7% higher than that at 25 °C. This enhancement in mechanical performance and cracking threshold limits reflected on an overall increase in the associated CSS, *S_r_*, and *H (t)* values and an overall decrease in *b*-values as listed in Table 10 and Table 11.

For instance, the AE parameters associated with the onset of the first detected micro-crack for mixture M4 (as shown in Table 10) witnessed an average increase of 21.8% in the cumulative number of hits, 27.6% in CSS, 38.6% in *H (t)*, and 9.4% in *S_r_* and an average decrease of 36.3% in *b*-values as a result of cooling down samples from 25 °C to −20 °C. The same pattern was noticed until the onset of the macro-cracking stage for the same mixture, as shown in Table 11. For instance, till the onset of the first macro-crack, there was an average increase of 11.2% in the cumulative number of hits, 8% in CSS, 11.9% in *H (t)*, and 18.3% in *S_r_* and an average decrease of 61% in *b*-values when testing samples at −20 °C compared to 25 °C. This pattern in the collected AE parameters is attributed to the increase in flexure resistance that made the mixtures resist higher loads at −20 °C.

### 6.3. Effect of W/C Ratio on the Behavior of AE Waves Emitted at −20 °C

Concrete mixtures with different microstructures generate different acoustic activity when they undergo cracking. Hence, mixtures M1 and M2 with different w/b (0.55 and 0.4, respectively) were included to evaluate the effect of changing the microstructure due to turning a huge amount of water into ice at −20 °C. Increasing the w/b was found to significantly decrease compressive strength, flexure strength, the load and time threshold of the micro-and macro-cracks’ onset, and the total AE activity recorded till the first micro-crack and till failure (which means less crack formation) when the samples were tested at 25 °C. Meanwhile, this reduction effect in structural performance and AE activity was not such noticeable when tests were performed at −20 °C.

For instance, increasing the w/b from 0.4 (M2) to 0.55 (M1) was a companied by a reduction of 9.2% in compressive strength at 25 °C compared to only 3.3% at −20 °C, and by 15.7% in flexure strength at 25 °C compared to only 5.6% at −20 °C (see Table 9). In addition, there was a decrease by 13.3% in micro-crack onset load at 25 °C compared to 9.1% at −20 °C, and the first micro-crack formation took 23 s less at 25 °C (for M1 compared to M2) compared to only 15 s less at −20 °C (refer to Table 10). Furthermore, till the detection of the first micro-crack, increasing the w/b from 0.4 (M2) to 0.55 (M1) resulted in a decrease of 37.2% and 25.7% in the collected number of hits, 15.1% and 13.8 in the CSS, 15.1% and 13.8 in the *H (t)*, and 15.1% and 13.8 in the *S_r_* at 25 °C and −20 °C, respectively. This reduction in the total collected AE activity and micro-crack onset load and time could be attributed to the reduction in the structural performance along with increasing w/b, which did not allow many cracks to appear.

### 6.4. Effect of Adding Fibers on the Behavior of AE Waves Emitted at −20 °C

To evaluate AE monitoring sensitivity to fiber pullout and breakage inside concrete mixtures, a non-fibrous control mixture (M3) and another four fibrous mixtures with different fiber lengths, types, and contents (M4–M7) were developed and tested. Overall, there was an enhancement in the flexural performance along with adding different types of fibers accompanied by an increase in the AE activity regardless of samples’ temperature during the test. For instance, compared to mixture M3, adding 0.2% of 19 mm synthetic polypropylene fibers increased the flexure strength by 5.6% when samples were tested at −20 (see Table 9) and an increase of 24% in the number of hits, 28.1% in CSS, 9.8% in *H (t)*, and 7.1% in *S_r_* for the total AE events recorded till the onset of the first micro-crack (as shown in Table 10).

**Figure 5:**
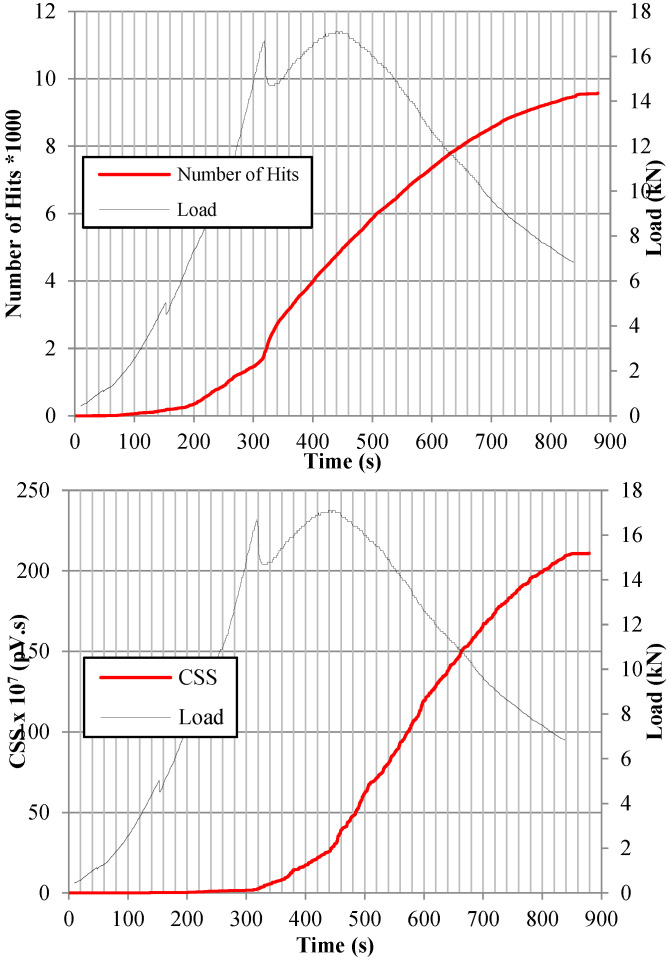

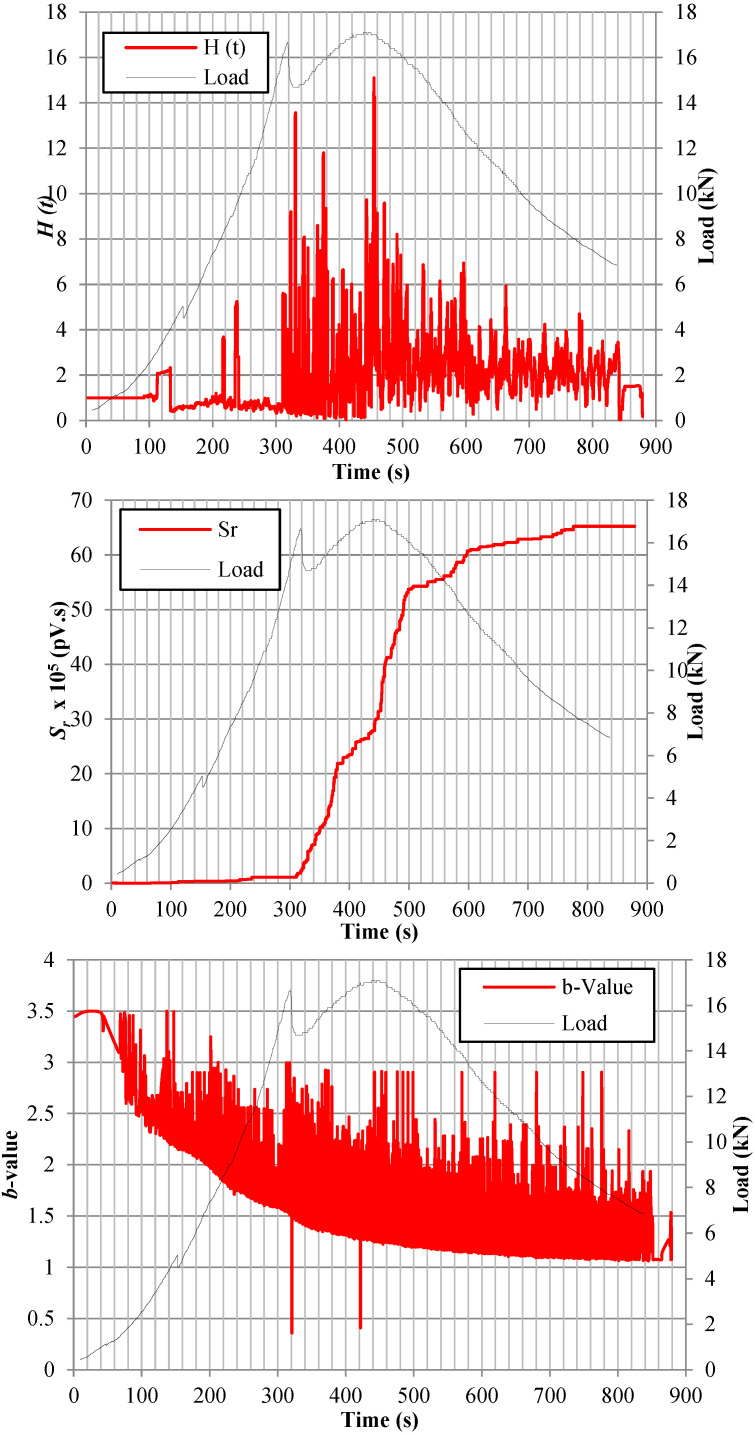
Flexure loading test along with AE parameter data, which were recorded by sensor-2 for sample-2 mixture M550-1%SynF38 tested at 25 °C.

This behavior could be attributed to the extra flexural strength added via including fibers and the mixtures that allowed more time and resistance for crack formation. In addition, fiber breakage and slippage could have caused extra acoustic activity that was recorded by the AE sensors. It is worth highlighting that for mixture M7 with 1% synthetic polypropylene fibers, there was a post macro-cracking load resistance and softening behavior, as shown in Figure 5. During that stage, the monitoring system recorded a high AE activity that could be caused by fiber pullout and breakage that may have induced extra acoustic waves. For instance, as shown in Figure 5, sample-2 in mixture M7 tested at 25 °C reached 16.7 KN till the first macro-crack and there was a slight drop in the resistance till the fibers started to hold back the load by itself and reached 17.1 KN, then shifted to the softening phenomenon. After reaching the macro-cracking load (16.7 kN) the total collected number of hits increased from 1938 to 9815, CSS increased from 4 × 10^7^ to 14.93 mV.s, *H (t)* kept fluctuating with an overall increasing pattern, and *S_r_* increased from 3 × 10^5^ to 16.93 × 10^5^ mV.s.

### 6.5. Effect of Fiber Type on the Flexural Performance and Behavior of AE Waves at −20 °C

Two different materials of fibers were included in the study: polypropylene synthetic in mixture M5 and steel in mixture M7. The steel fibers were supported to be more beneficial terms of enhancing flexure resistance with much more noticeable AE activity over polypropylene synthetic fiber mixtures at both temperature conditions. For instance, mixture M7 witnessed the onset of the first detected micro-crack after 207 s at load 8.13 KN compared to 112 s at load 6.23 KN for mixture M5 at 25 °C, as shown in Table 10. In addition, till the onset of the first micro-crack in mixture M7, the total collected AE parameters were 28.2% more number of hits, 23.9% more CSS, 26.8% more *H (t)* values, 8.6% more *S_r_* values, and 57% more in *b*-values in mixture M7 compared to M5 when both were tested at 25 °C. The increased AE activity could be attributed to the enhancement in the flexural performance.

It is worth noting that there was not any noticeable pattern in changing the values of wave signal’s amplitude along with changing fiber materials in both temperatures (25 °C and −20 °C), which eliminates any potential attenuation effect that could be proposed specifically in the case of polypropylene fibers considering its acoustic absorption capacity as a material. For instance, till the onset of first micro-crack, the wave signal amplitude detected by both channels was 80 and 81 dB in mixture M5 and 80 and 81 in mixture M7 when both were tested at 25 °C, as shown in Table 10.

### 6.6. Effect of Fiber Content on the Behavior of AE Waves Emitted at −20 °C

Fibers are mainly added to concrete mixtures to make it damaged by many narrow cracks, hence eliminating the undesired catastrophic failure. Hence, high acoustic activity is anticipated to be recorded during the flexure test. As a result, mixture M6 was included with 1% polypropylene synthetic fibers compared to only 0.2% fiber content at mixture M5 to monitor the AE activity, specifically the signal amplitudes values at both temperatures conditions. As shown in Table 11, although increasing the fiber content should increase the flexure performance, reaching such a high fiber content of 1% was found to decrease the flexure performance at both temperatures. For instance, the first macro-crack was detected at 37.19 KN after 294 s in mixture M6 compared to 42.34 KN after 304 s in mixture M5 when both were tested at −20 °C. Along with the reduction in flexure performance when increasing fiber dosage, the AE activity increased and mixture M6 experienced total hits of 2296; CSS of 5.43 × 10^7^ pV.s; and *H (t)* of 8.12, *S_r_* of 9.41 × 10^5^ mV.s, which were more than the AE data recorded for mixture M5 that were total hits of 2141, CSS of 5.37 × 10^7^ pV.s, *H (t)* of 7.66, and *S_r_* of 8.03 × 10^5^ mV.s.

These results could be attributed to the fact that increasing the fiber content over a certain value will not make a homogeneous mixture. Hence, the structural performance will degrade. In addition, the extra dosage of fibers will yield more acoustic activity due to the increased breakage and pull-out activity. These results support the conclusion that AE monitoring could be considered as a crack monitoring tool but not a structural performance evaluation tool. This conclusion matches the fact that the collected signals are related to the cracking activity of a certain mixture.

### 6.7. Effect of Fiber Length on the Behavior of AE Waves Emitted at −20 °C

Fiber length affects the failure mode in FRC mixtures. For instance, shorter fibers are anticipated to be subjected to pull-out. Meanwhile, longer fibers are anticipated to be broken. Hence, two FRC mixtures (M4 and M5) with two different Syn-P lengths, 19 mm and 38 mm, respectively, were included to investigate if there was any difference in the behavior of the acoustic activity developed in both mixtures. As shown in Table 9, mixture M5 with longer fibers showed a better flexural resistance with a delayed first visible micro-crack at both temperatures. Meanwhile, there was a lower AE activity detected in mixture M5 till failure. For instance, mixture M5 witnessed 19.05 MPa flexural strength and 6.23 MPa micro-crack threshold, which were 3% and 15.6%, respectively, higher than that of mixture M4 at −20 °C. In addition, mixture M5 witnessed 11.7% less number of hits, 5% less CSS, 19.1% less *H (t)* values, 2% less *S_r_* values, and 73.1% higher *b*-values than mixture M4 till failure at −20 °C (see Table 9, Table 10 and Table 11). The increased AE activity detected in mixture M4 could be attributed to the increased fiber pull-out occurrence due to the insufficient length of fibers. Meanwhile, mixture M5 has fibers with enough development length that they developed less acoustic activity. This observation confirms the conclusion that the AE technique is a non-destructive structural health monitoring technique, not a structural performance evaluation tool.

### 6.8. Classifying Micro- or Macro-Cracking Levels

#### 6.8.1. AE Intensity Analysis Parameter-Based Chart

**Figure 6:**
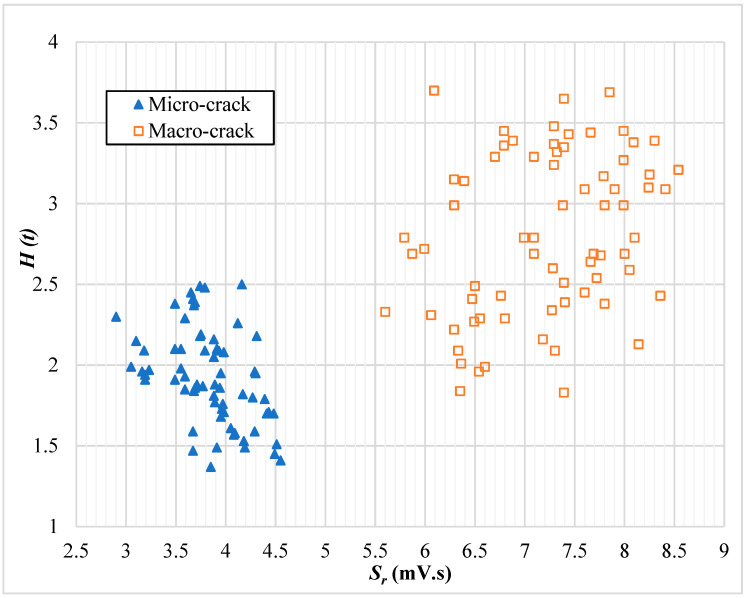
Crack level classification chart based on intensity analysis parameters.

Upon the results of the previous sections, intensity analysis parameters (*H (t)* and *S_r_*) were found to change along with crack progression. Hence, analyzing the variation of the values of both parameters was investigated in this study to classify the AE detected hits, whether associated with micro- or macro-cracks. The average intensity analysis parameters were taken from the two sensors, plotted on the chart shown in Figure 6, and correlated to the counterpart cracking level. Surprisingly, the dots plotted in the chart (Figure 6) clustered in two zones according to the cracking stage that they were recorded in. The values of *H (t)* and *S_r_* were found to have specific ranges depending on the crack source type. For instance, the ranges of the values of *H (t)* and *S_r_* were 1.37–2.55 and 2.89–4.61 × 10^4^ (pV.s), respectively, for the AE events that were detected during the micro-cracking stage (Table 10). Meanwhile, these ranges were 1.83–3.73 and 5.61–8.55 × 10^4^ (mV.s) for *H (t)* and *S_r_*, respectively, at the macro-cracking stage (Table 11). The chart in Figure 6 can further be used to estimate the cracking stage in a specific flexure test using the AE monitoring outputs. For instance, if the *H (t)* and *S_r_* values of a specific AE event were found to be 3.5 and 7.0 × 10^4^ (pV.s), respectively, then macro-cracks are anticipated at the time these signals were recorded. This chart has the potential to be a step on the way to develop cracking level classification criteria based on the AE intensity analysis parameters.

#### 6.8.2. RA Analysis-Based Chart

**Figure 7:**
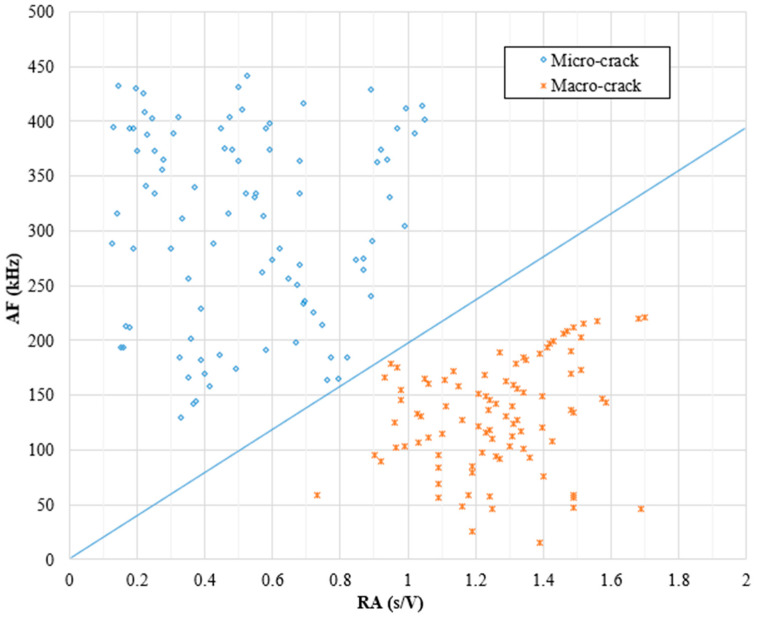
Crack level classification chart based on results of RA and AF analysis.

The RA analysis was mainly used in previous studies to classify the cracks developed during flexure tests into shear or tensile cracks. Upon identifying the AE events collected during the flexure tests, whether they were associated with micro- or macro-cracks, a trial was made to utilize the RA analysis to characterize the AE hits also. Alternatively, Figure 7 shows the values of RA vs. AF for all tested mixtures to find a specific pattern for micro- and macro-crack-related hits. It can be observed from Figure 7 that micro-crack events have relatively higher AF values and lower RA values. Meanwhile, macro-crack events have relatively higher RA values and lower AF values. In both charts, both point clusters were separated by a straight line (diagonal threshold) with a slope value “M” that was determined as indicated in Section 5. The M-values were 1.94 × 10^−3^ s/V/kHz. These results confirmed the effectiveness of the RA vs. AF analysis for classifying the crack level in all samples regardless of the mixture type or testing temperature. It should be kept in mind that the obtained M-values are exclusively related to these mixtures, loading type, and loading configuration.

## 7. Conclusions

This study investigated the properties of the acoustic emission waves accompanying cracking events in FRC mixtures under flexure at different temperatures (25 °C and −20 °C) along with visual inspection results. Three parameter-based AE analysis were implemented to develop a criterion regarding the pattern of the AE waves emitted during flexure test along with crack formation. The following findings were reached based on the current study:

The four AE parameter-based analyses utilized in this study were intensity, RA, *b*-value, and *Ib*-value analyses, which have the potential to highlight the cracking activity in the listed FRC mixtures at room and subfreezing temperatures (25 °C and −20 °C).

1. The initiation of micro- and macro-cracking coincided with a significant jump in the CSS, *H (t)*, and *S_r_* curves and an extreme dip in the *b*-value curve, while the bellows of the *Ib*-value curve fluctuation became noticeably concentrated only prior to macro-crack onset with no specific pattern change prior to micro-crack initiation.
2. Flexural performance was enhanced, and time and load thresholds of micro- and macro-cracks increased when the samples were cooled down and tested at −20 °C, significantly in mixtures with higher W/B, longer fibers, and lower fiber content. This improvement was also associated with higher AE activity in the form of increased CSS, *S_r_*, and *H (t)* values and lower *b*-values.
3. The AE activity monitoring and analysis detected the onset of micro-cracks when the loads reached approximately 27–36% and 33–41% of the failure loads when samples were tested at 25 °C and −20 °C, respectively.
4. Changing the concrete mixture design parameters, including the w/b ratio as well as fiber type, content, and length, resulted in a noticeable effect on the flexural behavior and the AE activity of the tested mixtures at both temperatures (25 °C and −20 °C).
5. The intensity analysis parameters (*H (t)*, and *S_r_*) were found to have specific ranges according to the associated crack type. Subsequently, a micro- and macro-crack classification chart was developed and presented to characterize the crack type based on the values of the *H (t)* and *S_r_* for the accompanying AE event.
6. Micro-crack events were found to have relatively higher AF values and lower RA values. Meanwhile, macro-crack events have relatively higher RA values and lower AF values. Consequently, an RA analysis-based chart was developed and presented to classify the recorded AE events whether correlated with micro- or macro-crack activity in the tested specimens. A diagonal line with a slope M = 194.44 × 10 ^−3^ kHz/s/V was found to split the AE events based on the values of RA and AF.
7. The micro- and macro-crack classification charts developed can be used to evaluate the flexural behavior of FRC at low temperatures within the range of the studied parameters in this study. Future research is needed to refine and assess the effectiveness of the developed charts in the assessment of actual concrete structures exposed to cold temperatures and monitored with AE sensors.

## Figures and Tables

**Table 1:** Concrete mixtures composition.

Mixture No.	Mixture Type	Cement (kg/m^3^)	MK (kg/m^3^)	FA (kg/m^3^)	C/F	W/B	C.A. (kg/m^3^)	F.A. (kg/m^3^)	Water Content (kg/m^3^)	V_f_ %	HRWRA (kg/m^3^)
M1	M500-0.55w/b	500	-	-	0.7	0.55	606.15	865.83	275	-	1.43
M2	M500-0.4w/b	500	-	-	0.7	0.4	686.57	981.21	200	-	2.17
M3	M550-control	275	110	165	0.7	0.4	619.67	885.84	220	-	3.51
M4	M550-SynF19	275	110	165	0.7	0.4	619.67	885.84	220	0.2	4.42
M5	M550-SynF38	275	110	165	0.7	0.4	619.67	885.84	220	0.2	4.69
M6	M550-1%SynF38	275	110	165	0.7	0.4	619.67	885.84	220	1	5.76
M7	M550-SF35	275	110	165	0.7	0.4	619.67	885.84	220	0.2	4.66

Note: 1 kg/m^3^ = 0.06243 lb/ft^3.^

**Table 2:** Typical features of steel and polypropylene fibers.

Fiber Type	Material	Length (mm)	Diameter (mm)	Specific Gravity	Tensile Strength (MPa)	Fiber’s Shape
SF-35	Steel	35	0.55	7.85	1150	single hooked-end
SynF-19	polypropylene	19	0.66	0.91	300	straight
SynF-38	polypropylene	38	0.64	0.91	515	straight

Notes: 1 mm = 0.039 in.; 1 MPa = 145 psi.

**Table 3:** Chemical composition of all cementitious materials used in mass percent (%).

Chemical Properties F%	Cement	MK	Fly Ash
SiO_2_	19.63	51–53	51
Al_2_O_3_	5.48	42–44	23
Fe_2_O_3_	2.37	<2.2	12
FeO	--	--	--
TiO_2_	--	<3.0	--
C	--	--	--
Cr_2_O_3_	--	--	--
MnO	--	--	--
P_2_O_5_	--	<0.21	--
SrO	--	--	--
BaO	--	--	--
SO4	--	<0.5	--
CaO	62.43	<0.2	5
MgO	2.47	<0.1	--
Na_2_O	--	<0.05	--
C_3_S	52.33	--	--
C_2_S	16.82	--	--
C_3_A	10.51	--	--
C_4_AF	7.23	--	--
K_2_O	--	<0.40	--
L.O.I	2.04	<0.50	--

**Table 4:** Physical properties of all cementitious materials used.

	Cement	MK	Fly Ash
Specific Gravity	3.15	2.5	2.38
Blaine Fineness (m^2^/kg)	410	19,000	420

**Table 5:** Settings of the AE acquisition system.

AE Setup Parameters	Values
Threshold	40 dB_AE_
Sample rate	1 MSPS
Pre-trigger	256 µs
Length	1k points
Preamp gain	40 dB
Preamp voltage	28
Analog filter	1–50 kHz
Digital filter	100–400 kHz
Peak definition	200 µs
Hit definition time	800 µs
Hit lockout time	1000 µs
Maximum duration	1000 µs

**Table 6:** Acceptance limits for AE amplitude-duration filter.

Amplitude Range (dB)	Duration (µs)		Amplitude Range (dB)	Duration (µs)	
	Lower	Upper		Lower	Upper
40 ≤ A < 45	0	400	60 ≤ A < 65	300	1000
45 ≤ A < 48	0	500	65 ≤ A < 70	500	2000
48 ≤ A < 52	0	600	70 ≤ A < 80	1000	4000
52 ≤ A < 56	0	700	80 ≤ A < 90	2000	7000
56 ≤ A < 60	100	800	90 ≤ A < 100	3000	10,000

**Table 7:** The K-parameter used to calculate the *H (t)* for concrete structures.

Number of Hits, N	≤50	51–200	201–500	≥501
K	0	N-30	0.85N	N-75
J	0	50	50	50

**Table 8:** Examples of M-values obtained in concrete-based specimens tested under a four-point flexure test.

Authors	Mixture Type	Dimensions (mm)	Loading Type	Max RA (ms/V)	Max A-FRQ (kHz)	M (s/V/kHz)
Soulioti, Barkoula [48]	Steel-fiber-reinforced concrete (SFRC)	100 × 100 × 100	Monotonic	6	65	0.09
Aggelis [49]	Plain concrete	100 × 100 × 400	Monotonic	15	550	0.027
Shahidan, Bunnori [50]	Steel-reinforced concrete (RC)	150 × 250 × 1900	Cyclic	150	150	1
Behnia, Chai [51]	RC, SFRC, polypropylene FRC	200 × 250 × 2500	Cyclic	5 × 10^−3^	400	12.5 × 10^−6^
Prem and Murthy [52]	RC	100 × 200 × 1500	Monotonic	200	200	1

**Table 9:** A summary of test results of the developed mixtures, tested at 25 °C and −20 °C.

Mixture No.	Mixture Type	Testing Temperature	Compressive Strength (MPa)	Maximum Flexure Load (KN)	Flexure Strength (MPa)
M1	M500-0.55w/b	25 °C	61.16	23.64	10.64
		−20 °C	76.68	32.75	14.74
M2	M500-0.4w/b	25 °C	66.79	27.35	12.31
		−20 °C	79.18	34.61	15.57
M3	M550-control	25 °C	74.12	31.14	14.01
		−20 °C	89.91	39.85	17.93
M4	M550-SynF19	25 °C	78.63	32.97	14.84
		−20 °C	90.77	43.19	18.54
M5	M550-SynF38	25 °C	76.16	33.76	15.19
		−20 °C	88.28	43.34	19.05
M6	M550-1%SynF38	25 °C	51.75	30.89	13.90
		−20 °C	63.67	37.19	16.74
M7	M550-SF35	25 °C	78.74	35.64	16.04
		−20 °C	89.59	44.73	19.68

**Table 10:** AE parameters collected at the first visible micro-crack onset at 25 °C and −20 °C.

Mixture No.	Mixture Type	Testing Temperature	Signal Amplitude (dB)		Number of Hits		CSS (pV.s) × 10^6^		*H (t)*		*S_r_* × 10^4^ (mV.s)		*B*-Value		First Micro-Crack Load (KN)	First Micro-Crack Time (s)	
			CH-1	CH-2	CH-1	CH-2	CH-1	CH-2	CH-1	CH-2	CH-1	CH-2	CH-1	CH-2		AE Analysis	Visually Noticed
M1	M500-0.55w/b	25 °C	81	83	89	76	1.77	1.69	1.77	1.66	4.32	4.22	3.43	3.64	3.76	61	63
		−20 °C	80	81	106	98	1.98	1.83	1.93	1.84	4.53	4.36	2.86	2.97	4.11	79	80
M2	M500-0.4w/b	25 °C	79	81	112	121	2.05	1.99	2.05	1.98	4.59	4.65	3.23	3.08	4.26	84	86
		−20 °C	80	82	124	132	2.16	2.24	2.19	2.13	4.72	4.76	2.57	2.75	4.89	94	94
M3	M550-control	25 °C	79	80	132	139	2.28	2.21	2.25	2.32	4.81	4.79	2.32	2.19	5.33	89	90
		−20 °C	81	79	146	163	2.49	2.55	2.36	2.77	4.96	4.88	2.04	2.13	5.81	103	105
M4	M550-SynF19	25 °C	83	81	178	183	2.76	2.83	2.47	2.41	5.07	5.13	0.71	0.80	6.92	114	121
		−20 °C	84	82	191	203	2.99	3.01	2.57	3.04	5.25	5.41	0.46	0.51	7.21	131	135
M5	M550-SynF38	25 °C	80	81	155	163	2.48	2.55	2.29	2.39	4.81	4.91	1.49	1.56	5.89	97	104
		−20 °C	82	79	169	174	2.63	2.51	2.43	2.66	4.96	5.05	1.84	1.71	6.23	112	117
M6	M550-1%SynF38	25 °C	84	83	184	192	2.69	2.73	2.59	2.65	4.97	5.03	1.55	1.63	6.34	109	118
		−20 °C	81	80	183	201	2.81	2.88	2.77	2.89	5.07	5.18	1.31	1.22	6.69	123	131
M7	M550-SF35	25 °C	80	81	203	209	3.09	3.16	2.94	3.03	5.29	5.33	0.53	0.67	7.64	179	187
		−20 °C	82	80	217	226	3.21	3.24	3.07	3.21	5.41	5.52	0.31	0.44	8.13	207	212

**Table 11:** AE parameters collected at the onset of the macro-cracking stage at 25 °C and −20 °C.

Mixture No.	Mixture Type	Testing Temperature	Signal Amplitude (dB)		Number of Hits		CSS (pV.s) × 10^4^		*H (t)*		*S_r_* (mV.s)		*B*-Value		Macro Crack Load (KN)	Macro Crack Time (s)
			CH-1	CH-2	CH-1	CH-2	CH-1	CH-2	CH-1	CH-2	CH-1	CH-2	CH-1	CH-2		
M1	M500-0.55w/b	25 °C	79	81	1424	1389	3.95 × 10^7^	3.73 × 10^7^	4.51	4.22	5.91 × 10^5^	5.85 × 10^5^	3.92	2.17	23.64	206
		−20 °C	81	83	1651	1589	4.19 × 10^7^	4.02 × 10^7^	5.03	4.89	6.27 × 10^5^	6.13 × 10^5^	3.19	3.31	32.75	248
M2	M500-0.4w/b	25 °C	80	79	1756	1813	4.56 × 10^7^	4.37 × 10^7^	5.24	5.33	6.68 × 10^5^	6.51 × 10^5^	2.97	3.04	27.35	251
		−20 °C	82	83	1844	1901	4.78 × 10^7^	4.69 × 10^7^	5.76	5.83	7.29 × 10^5^	7.18 × 10^5^	2.72	2.81	34.61	263
M3	M550-control	25 °C	81	84	1969	2006	4.88 × 10^7^	4.93 × 10^7^	5.91	5.79	7.46 × 10^5^	7.39 × 10^5^	2.46	2.55	31.14	277
		−20 °C	79	82	2034	2103	5.16 × 10^7^	5.27 × 10^7^	6.15	6.37	7.67 × 10^5^	7.55 × 10^5^	1.94	2.03	39.85	286
M4	M550-SynF19	25 °C	81	83	2307	2341	5.39 × 10^7^	5.46 × 10^7^	8.04	8.33	7.88 × 10^5^	7.73 × 10^5^	0.86	0.99	32.97	306
		−20 °C	83	81	2463	2391	5.62 × 10^7^	5.68 × 10^7^	8.38	9.12	8.09 × 10^5^	7.91 × 10^5^	0.23	0.29	43.19	327
M5	M550-SynF38	25 °C	79	81	2068	2034	4.96 × 10^7^	4.88 × 10^7^	6.89	7.04	7.13 × 10^5^	7.34 × 10^5^	1.76	1.86	33.76	281
		−20 °C	82	79	2109	2141	5.29 × 10^7^	5.41 × 10^7^	7.46	7.66	7.72 × 10^5^	8.03 × 10^5^	1.13	1.08	42.34	309
M6	M550-1%SynF38	25 °C	81	82	2237	2209	5.16 × 10^7^	5.09 × 10^7^	7.32	7.68	8.65 × 10^5^	8.86 × 10^5^	0.89	0.94	30.89	266
		−20 °C	80	83	2343	2296	5.33 × 10^7^	5.43 × 10^7^	7.91	8.12	9.23 × 10^5^	9.41 × 10^5^	0.66	0.59	37.19	294
M7	M550-SF35	25 °C	79	81	2591	2678	5.89 × 10^7^	5.77 × 10^7^	9.11	9.73	11.21 × 10^5^	11.37 × 10^5^	0.37	0.41	35.64	394
		−20 °C	83	81	2719	2788	6.13 × 10^7^	6.33 × 10^7^	9.87	10.13	12.05 × 10^5^	11.86 × 10^5^	0.18	0.23	44.73	434

## Data Availability

No new data were created or analyzed in this study. Data sharing is not applicable to this article.
